# Panic Attack during Elective Gastrointestinal Endoscopy

**DOI:** 10.1155/2011/162574

**Published:** 2011-10-04

**Authors:** Charalampos Mitsonis, Nikolaos Dimopoulos, Marianna Zavrou, Vassiliki Psarra, Christos Giofkos, Christos Fiorakis, Athanasios Dimitriadis, Dimitrios Valavanis, Eleni Vousoura, Iannis Zervas, Efstathios Papavassiliou

**Affiliations:** ^1^18th Psychiatric Department, Psychiatric Hospital of Attiki “Dafni”, 374 Athinon Avenue, 12462 Chaidari, Greece; ^2^6th Psychiatric Department, Psychiatric Hospital of Attiki “Dromokaition”, Iera Odos 243, 12461 Athens, Greece; ^3^3rd Psychiatric Department, Psychiatric Hospital of Attiki “Dafni”, 374 Athinon Avenue, 12462 Chaidari, Greece; ^4^F. P. Ps. Program of Psychology, Zografou University Campus, 15703 Athens, Greece; ^5^First Department of Psychiatry, Eginition Hospital, Athens University Medical School, Vas. Sofias 72-74, 11528 Athens, Greece; ^6^Department of Gastroenterology, Amalia Fleming Hospital, 25 Martiou 14, 15127 Melissia, Greece

## Abstract

*Background*. Esophagogastroduodenoscopy (EGD) and colonoscopy (CS) can evoke anxiety, embarrassment, and discomfort. These concerns can culminate in panic attacks, which may traumatize patients and significantly decrease their compliance to the procedure. The objective of this study was to evaluate the relationship between preendoscopic anxiety and the possibility of a panic attack during an elective gastrointestinal endoscopy (EGE). *Methods*. The study population comprised of 79 Greek outpatients. The examination was carried out without the use of conscious sedation. Patients' anxiety levels were assessed before the procedure using the Greek version of the Spielberger State-Trait Anxiety Inventory (STAI-Y). *Results*. Seventy-nine patients were enrolled: 45 EGD and 34 CS. Females had higher state and trait anxiety levels than males (48.14 ± 7.94 versus 44.17 ± 7.43, *P* < 0.05; and 43.68 ± 6.95 versus 39.86 ± 7.46, *P* < 0.05). Patients who experienced panic attack had significantly higher levels of both trait and state anxiety, compared to those who were panic-free. There was no significant relationship between panic attacks and sex or type of procedure. *Conclusions*. Patients who experience panic attacks during endoscopic procedures appear to have significantly higher anxiety levels before the procedure. Administering the STAI questionnaire prior to the endoscopy seems to be a useful screening method for vulnerable patients.

## 1. Introduction

Esophagogastroduodenoscopy (EGD) and colonoscopy (CS) are routinely performed diagnostic procedures that can be carried out without sedation [1]. However, patient acceptability of endoscopic procedures may be reduced due to fears of embarrassment, discomfort, and worry [2–6]. These concerns are often accompanied by an increase in patients' anxiety levels, which during the endoscopic procedure may culminate in a panic attack (i.e., episodes of excessive anxiety comprised of numerous somatic complains, fear of dying, and derealization) [7]. 

Wait times for hospital screening colonoscopy have increased dramatically in recent years, and patients are often referred directly for endoscopy without previously meeting with the performing practitioner. As a result, patients rarely have the opportunity to discuss their concerns about endoscopic procedures with health care providers or the personnel involved in the performed procedure [8]. However, failure to accurately detect and address patients' anxiety before the endoscopic examination may adversely impact their willingness to undergo such procedures [9]. Moreover, experiencing a panic attack may decrease patient's compliance during the endoscopic procedure. 

Several effective ways of reducing preprocedural anxiety exist. The standard procedure for decades has been conscious sedation along with the administration of benzodiazepines. These practices, although usually safe, may result in increased procedural costs and medical complications [10, 11]. An increasingly used alternative is propofol, used alone or in combination with midazolam or narcotics, which has been shown to result in shorter recovery time, greater patient tolerance, and high physician satisfaction [12]. However, most routine procedures do not merit sedation. In Europe, almost 3/4 of endoscopies are carried out without sedation [13]. Moreover, there are efficacious nonmedical methods of addressing patient anxiety, such as psychological interventions, relaxation, and music [14–16].

The aim of this study was to evaluate the relationship between preendoscopic anxiety and the possibility of a panic attack during elective gastrointestinal endoscopy (EGE).

## 2. Methods

Eligible subjects included Greek outpatients older than 18 years of age undergoing diagnostic upper endoscopy or colonoscopy performed by an experienced gastroenterologist. Only patients with no history of prior upper or lower endoscopy were included in the sample, as it has been found that first endoscopy patients experience significantly higher levels of anxiety than repeat endoscopy patients [17]. Subjects had either previously consulted with the performing endoscopist or referred directly for endoscopy without prior consultation through an “open access” endoscopy program. Hospital inpatients were excluded because they represented a small subset of patients undergoing potentially therapeutic procedures and often experienced significant stress related to comorbid conditions. Patients who had history of any clinically relevant psychiatric disease and patients who systematically used psychotropic medication or illicit drugs were also excluded. 

Sample size estimation was based upon prior studies using STAI-Y in patients undergoing endoscopy [4]. The sample size needed to reveal a correlation between trait and state anxiety (and the subsequent possibility of a panic attack) was calculated using data from Cohen [18]. In order to detect a moderate correlation (*r* = 0.30) at 0.05 level of statistical significance, 80 participants were needed to obtain a statistical power of 0.78 and a type II error probability of 0.22.

The study was approved by the Institutional Review Board at Amalia Fleming General Hospital, and informed consents were obtained from the subjects. Before the procedure, patients were informed in detail that the endoscopy will be carried out with topical pharyngeal anesthesia alone (100 g/L spray), without the use of conscious sedation. 

Subsequently and after providing informed consent, patients completed the Spielberger State-Trait Anxiety Inventory Form Y (STAI-Y), Greek version [19, 20]. This a 40-item questionnaire that measures separately the relatively stable tendency for experiencing anxiety (trait anxiety) and the level of anxiety experienced at the time of assessment (state anxiety). Questions are rated on a 4-point intensity scale, and higher scores indicate higher levels of anxiety. This measure was selected on the basis that it allows for a comparative assessment of situational anxiety and predisposition to anxiety as a personality trait. Moreover, it has good validity (Cronbach's *α* = 0.93) and reliability (*r* = 0.96 and *r* = 0.98, for state and trait, resp.) in the Greek population [20] and has been previously used for a variety of medical procedures, such as endoscopic [4], obstetric [21], and general surgical [22]. 

After completing the questionnaire, patients were gowned for endoscopy and brought to the endoscopy suite. Both endoscopic procedures were carried out by the same gastroenterologist in the presence of a clinical psychiatrist who was responsible for identifying the presence of a panic attack during endoscopic procedure according to the criteria of the diagnostic and statistical manual of mental disorders (DSM-IV-TR). When a panic attack occurred during endoscopy, the examination was terminated and scheduled for subsequent time (3–10 days).

Data were expressed with mean and standard deviation. Normality of all data sets was determined using the Kolmogorov-Smirnov (KS) test. Differences between groups were examined using the *t*-test and the paired *t*-test. Additionally Pearson correlation was used as measure of association for quantitative variables. Statistical significance was set as *P* < 0.05.

## 3. Results

**Figure 1:**
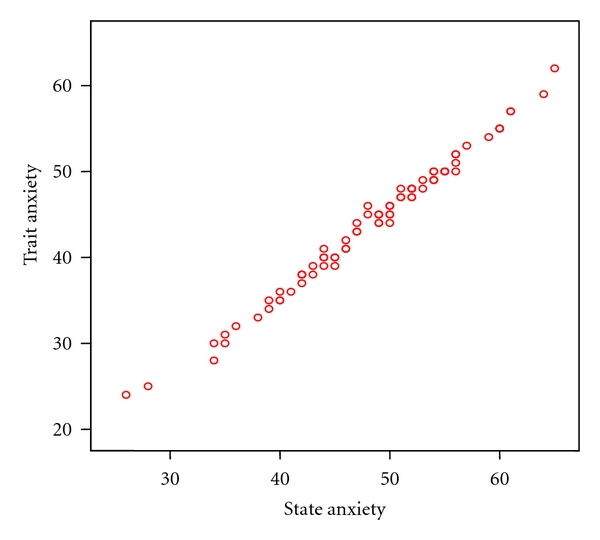
Correlation between trait and state anxiety.

**Figure 2:**
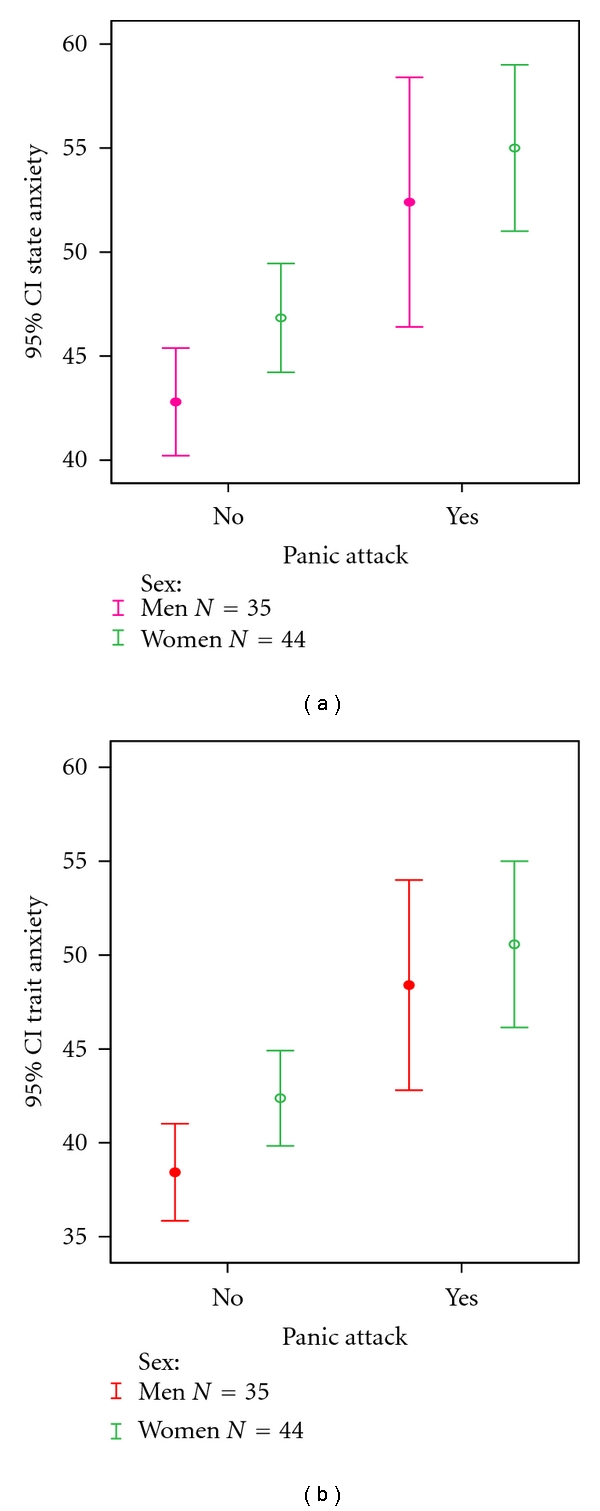
Mean and standard error for state and trait anxiety in patients with and without panic attack in EGE.

Eighty-eight patients met inclusion criteria, of which 79 agreed to participate in the study. Nine declined to participate for personal reasons. Females had higher levels of state (48.14 ± 7.94) and trait (43.68 ± 6.95) anxiety than males (44.17 ± 7.43 and 39.86 ± 7.46, resp.) (*P* < .05). Seven women and five men had a panic attack during the procedure (Table 1). A linear relationship was observed between state and trait anxiety (Pearson Correlation =  .89) (Figure 1). Patients who experienced panic attack during the examination had significantly higher trait (*P* < .001) and state (*P* < .001) anxiety levels than those who were panic-free (Figures 2(a) and 2(b)). EGD and CS induced similar preendoscopy anxiety (*P* > .05). There was no significant relationship between panic attacks and type of procedure. Panic attack during EGE was not influenced by sex.

## 4. Discussion

EGD and colonoscopy CS are commonly performed diagnostic procedures and considered routine by the medical community. However, patients' anxiety levels may significantly rise during such procedures, sometimes escalating to full blown panic. In our study, twelve out of 79 first endoscopic patients experienced a panic attack during the procedure. 

The main finding of our study is that a significant relationship was observed between panic attacks and both state and trait anxiety during endoscopy. Overall, patients undergoing diagnostic endoscopy showed modest increase in state anxiety; however, patients who experienced a panic attack had significantly higher trait and state anxiety levels than those who were panic-free. 

No significant relationship was observed between panic attacks and type of endoscopic procedure. Additionally, there was no significant relationship between panic attacks and patient sex although a slightly greater sensitivity was observed for women. Patients with the highest levels of anxiety in our sample experienced chest pain and sensations of shortness of breath, but none of them developed signs of myocardial ischaemia during the procedure. As Tønnesen et al. [6] have shown, the observed tachycardia is the result of a typical endocrine metabolic stress response. 

EGD and CS can be difficult procedures, especially when they are carried out without conscious sedation [23]. Endoscopy is considered by many patients as an invasive and painful procedure. Negative attitudes about endoscopy can decrease patients' willingness to undergo screening, and as such, it can be a significant barrier to care. Addressing patients' preprocedural attitudes about the examination is key to improving participation in colonoscopy [24].

In our study, patients were informed prior to the endoscopy that the procedure will be carried out with topical pharyngeal anesthesia alone, without the use of conscious sedation. Pompeo and Mineo [7] suggest that conversion to general anesthesia, even to pulmonary resection, is mainly caused by the presence of extensive fibrous pleural adhesions or the development of intractable panic attacks. Sedation with benzodiazepines facilitates the endoscopy, by helping patients rest during the procedure [25]; however, although sedation is the most widely employed method, it is not risk-free. 

 Recent studies stress the importance of discussing patients' anxiety and concerns before the procedure to avoid adverse outcomes [26, 27]; their findings suggest that provision of information is effective in reducing patients' anxiety and improving procedure tolerance. Based on these findings, patients should be informed in simple language about the type of procedure being performed, so that they can decide whether or not to undergo the proposed procedure. Following this information, patients should have the opportunity to express their feelings and concerns about the procedure and all its possible risks. One suggestion would be to show an educational video a week prior to the endoscopy, as it has been found that watching a video is associated with improvements in short-term knowledge [28, 29].

Several nonpharmacological interventions, such as relaxation music, educational material including videotapes, relaxation, and coping techniques, have been shown to effectively reduce anxiety levels and to prevent the emergence of panic attacks during endoscopy. Music played during endoscopic procedures may alleviate anxiety and improve patient acceptance of the procedure according to Bampton and Draper, Tam et al., and Costa et al. [14–16]. Medical staff should be aware of the positive effects of nondrug interventions and incorporate into pain management plans [30].

Further investigation incorporating cutoff points is recommended to identify the patients who are likely to experience panic attack and cannot tolerate EGE without sedation. STAI may be used as a prognostic tool of panic attacks during EGE and, thus, as a mean of early and successful management. 

In conclusion, endoscopy is an interventional procedure that can be stressful for some patients and may induce panic attacks. Patients who experience panic attacks during endoscopic procedures appear to have significantly higher anxiety levels before the procedure. Administering the STAI questionnaire prior to the endoscopy seems to be a useful screening method for the selection of vulnerable patients who may be in particular need for conscious sedation during elective GI endoscopy. Limitations of the study were the relatively small sample size and that as a study of unsedated endoscopy its findings may not be generalizable to patients who present with more severe clinical characteristics and require sedated endoscopy.

## Figures and Tables

**Table 1:** Demographic and clinical data of the patients.

	Males	Females
Patients (*n*)	35	44
Age (mean yrs)	57 (range 31–77)	54 (range 24–80)
EGD (*n*)	20	25
CS (*n*)	15	19
Trait anxiety ± SD	39.86 ± 7.46	43.68 ± 6.95*
State anxiety ± SD	44.17 ± 7.43	48.14 ± 7.94*
Panic attacks (*n*)	5	7

*n*: number, EGD: Esophagogastroduodenoscopy, CS: Colonoscopy, SD: Standard deviation.

*P < 0.05 versus males.
